# Comparison of Traditional Impression and 3D Ear Scanning Techniques, Earmold Comfort, and Audiology Clinical Implications: A Pilot Study

**DOI:** 10.3390/bioengineering10121431

**Published:** 2023-12-16

**Authors:** Yunfang Zheng, Karlina Didion, Nicole Ferguson

**Affiliations:** Department of Communication Sciences and Disorders, Central Michigan University, Mount Pleasant, MI 48859, USA

**Keywords:** traditional impression, 3D ear scanning, earmold, clinical implication

## Abstract

This study investigated clinical aspects of the traditional ear impression and 3D ear scanning techniques. Adult earmold-users and non-users participated in this study. The earmold-users also participated in the earmold comfort comparison study by wearing earmolds from both techniques, one set a week according to a randomized sequence. Multiple clinical aspects of both techniques according to the participants and audiology professionals were recorded. Results revealed a preference for the 3D-scanning technique, which was perceived as more comfortable although both techniques were perceived as safe. Although the earmolds might have issues from both techniques, there was no significant difference in the perception of earmolds. Experience with the specific technique can affect the responses from the professionals. Compared to the traditional technique, 3D-scans had higher fixed but less variable costs and procedure times. A special clinical case was included and indicated that 3D-scans could be an option for specific patients. This study led to a better understanding of the two techniques clinically. With increasing involvement of new technology and more young professionals joining the profession of audiology, 3D ear scanning could be a viable consideration for audiology practices.

## 1. Introduction

Hearing instruments have endured an amazing and extensive journey to arrive where they are today in both technology and appearance. The transformation from the acoustic era in which twelve-inch funnel-shaped ear trumpets were introduced, to today’s era of connectivity in which we have small, nearly invisible hearing aids is incredible [1,2,3,4,5]. Advancements in technology in the field of audiology are vast and ever changing; therefore, audiologists must continue training and education within these emerging areas in the field to provide the best care and ensure overall good quality of life for each patient. Ear impressions are a very important audiology technique to create custom-fit earmolds/hearing aid shells when necessary. They are molded to each individual’s ears to retain the hearing aids, deliver amplified sounds to the ear canal, provide a satisfactory acoustic seal of the ear canal, and more importantly, acoustically modify the gain and output of the hearing aid for a satisfactory fitting outcome [1,6,7]. Ear impressions have evolved from the traditional impression technique to newly developed 3D-scanning.

Ear impression techniques stem from materials used for impressions of teeth for dentistry, and the first ear impressions were made with plaster in 1890 [8]. Currently, there are three main types of impression materials—condensation-cured silicone, addition-cured silicone, and acrylic material [1,9]. All these materials include two parts which should be mixed well and then delivered into the ear via a syringe or an impression gun within 20 to 30 s, and cured in the ear for approximately 5 min [10,11]. Before the impression material is delivered into the ear, an otoblock must be placed just beyond the second bend of the ear canal to avoid possible damage to the tympanic membrane (TM) from the impression material and to ensure a good representation of the ear canal shape to result in a good fit of the earmold [11,12]. Otoscopic examination of the otoblock placement is important to ensure proper size and placement before inserting impression material. 

With traditional impression techniques, audiology professionals have provided many well-fit earmolds/custom hearing aids to different individuals to improve fitting outcomes. However, there are some limitations of this technique. First, the impression materials must have contact with the skin of the ear, so this technique cannot be used on individuals with sensitive skin, those prone to bleeding, those with otitis externa, active ear drainage, or multiple exostoses, etc. [13]. Second, without both the proper sizing and placement of the otoblock in addition to proper insertion of the impression material, complications or injury to the patient could occur. Studies have reported impression material passing the otoblock, causing TM perforations, impairment of the middle ear ossicles, cavity, or mastoid, or even affecting the inner ear [14,15,16,17,18]. Third, this technique is not clinically time efficient. The ear impressions are mailed to the manufacturer and then the earmolds are mailed back, which usually takes approximately two to three weeks. Fourth, there are possible ear impression defects during shipping, especially for those made from acrylic materials which will be affected by temperature and humidity and will shrink by 2–5% 48 h after the impression is formed [19,20]. The shrinkage rate is 0.1% and 0.5% for addition-cured and condensation-cured silicone materials, respectively [20,21].

In 2005, iScan, a three-dimensional scanner introduced by Siemens (Piscataway, NJ, USA) was used in the clinics to scan ear impressions made from the traditional technique and to create a digital image, which would then be emailed to the manufacturer to fabricate earmolds or custom hearing aids [9,22]. The iScan helped prevent impression defects during shipping and reduced the shipping time in half, and saved on shipping cost because there is no need to ship the impressions to the manufacturer. However, the limitations associated with impression production remained. In 2013, a 3D ear scanning system was launched by Lantos Technologies (Derry, NH, USA) [23]. It uses a handheld video scanner with a conforming membrane (CM) connected to the tip of the video scanner and filled with water. After insertion into the ear canal beyond the second bend, the CM expands to the shape of the ear canal and hundreds of images of the ear (concha, helix, and ear canal) are taken, from which it generates a 3D digital scan that is then sent to the manufacturer electronically. The CM will only expand radially not medially, which helps prevent any damage to the tympanic membrane and beyond. Similar to the iScan, this 3D ear scan presents the advantage in time efficiency and shipping cost. However, it still introduced a foreign body to the ear canal, which may limit its use on individuals mentioned above.

In 2018, Otoscan, a laser 3D ear scanning device, was launched by Natus (Middleton, WI, USA) [24]. It utilizes lasers (line and ring laser) without touching the skin to scan the ear and creates a 3D image of the ear, uploads to an online storage cloud (Otocloud) that can be accessed by both audiology professionals and the hearing instrument manufacturers for further earmold development [25,26]. This technique potentially eliminates the concerns around using the traditional impression technique, so it could be a viable option from the traditional technique.

Currently, there is no published research regarding how 3D ear scanning techniques compare to traditional ear impressions clinically. This pilot study intended to investigate the differences between the two techniques based on multiple clinical aspects, including comfort and safety during the impressions, the comfort of earmolds made by each technique, and clinic time and cost to provide insight regarding the clinical usage of the two techniques from the viewpoints of both the patients and audiology professionals. Two audiology professionals (one experienced and one young clinician) participated in this study to investigate possible experience effects on the two techniques. A special clinical case reported below provides an example for clinical consideration in choosing the appropriate impression method to help individual patients achieve desired clinical outcomes.

## 2. Methods

### 2.1. Subjects

Twelve adults participated in this study, six experienced earmold users (age = 53–68 years, mean = 61.7 years) and six non-earmold users (age = 21–25 years, mean = 23 years). Background information of the subjects with earmolds is provided in Table 1. All subjects were native English speakers with clear and healthy ear canals.

### 2.2. Audiology Professionals

Two audiology professionals administered the impression techniques. One is a licensed audiologist with 18+ years of experience in audiology including traditional impression technique. The other is a second-year audiology doctoral student, who started making ear impressions on patients after completing formal audiology training. Both audiology professionals had approximately one month of experience with the 3D-scanning technique.

### 2.3. Impression Technique Comparison

#### 2.3.1. Overall Procedure

Prior to the impression procedures, otoscopy was performed to ensure clear and healthy ear canals, bilaterally. All participants underwent traditional ear impressions as well as 3D ear scanning in a randomized sequence. The audiologist performed the techniques on the earmold users, while the audiology doctoral student performed them on non-earmold users. All participants completed a questionnaire (0–5 scale, 5 being the highest rating) after each ear impression was taken regarding feelings of comfort and safety as well as their preference between the two techniques. The audiology professionals also completed a questionnaire using the same 5-point scale regarding the techniques in four categories including comfort in administering the technique, ensuring the safety of patients, overall preference for technique, and clinical efficiency. Time spent on each procedure per ear was tracked by the investigator, including from the end of otoscopy to the end of impression material delivery (t1/T1) or the end of scan (t1/3D1), and from the end of otoscopy to the end of impression removal and inspection (t2/T2) or the end of scan inspection (t2/3D2). The cost of supplies for each technique was also tracked by the investigator.

#### 2.3.2. Ear Impression Procedures

**Figure 1:**
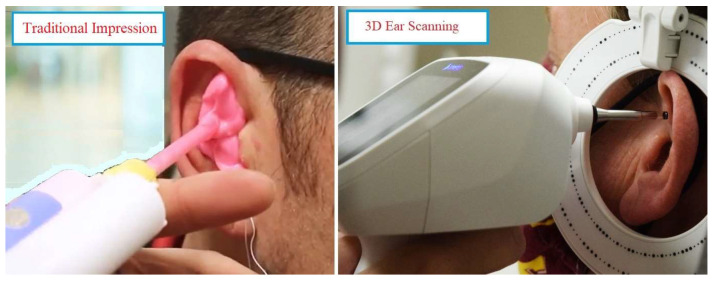
Traditional impression and 3D ear scanning techniques.

The traditional ear impression involved using the Westone S-50 impression gun (model D5) to deliver silicone material. The procedure comprised of otoscopy, placement of an otoblock just beyond the 2nd bend of the ear canal, injection of impression material, removal of the cured impression, and inspection of the ear. For 3D ear scanning, the Otoscan was employed. The process included otoscopy, scanning the ear to generate a 3D image, and inspecting the resulting 3D image. See Figure 1. The same technique was then applied to the other ear.

### 2.4. Earmold Comfort Comparison

To eliminate the experience effect, only the earmold users participated in this study section. All participants experienced earmolds made from both the traditional and 3D-scanning techniques. The earmolds were ordered with the same specifications as their existing earmolds fit their hearing aids (see Table 1).

Randomly selected, half of the subjects were fit with earmolds produced by the traditional technique and wore them for one week. The other half of subjects started week one with earmolds produced by 3D-scanning. After one week, all participants were fit with the other set of earmolds, then wore them for another week. Participants completed a questionnaire (0–5 scale, 5 being the highest rating) regarding their experience with each set of earmolds. The questionnaire was completed during the initial fit of each set of earmolds and after one week use of the earmolds. Subjective feedback from participants was recorded regarding the impression techniques and the earmolds created.

### 2.5. Special Clinical Case

A special clinical participant was included as this case presented medical necessity for the 3D-scanning technique. This patient had Down Syndrome with a history of a skin disorder in the ear canals, which caused the skin in the ear canals to consistently grow inward, leading to the closing of the ear canals and hence, causing a repetitive conductive hearing loss until the canals were surgically re-opened. This led to the need for use of hollow earmolds to force the ear canals to remain open. This patient had undergone an extensive history of traditional ear impressions being unable to obtain a deep enough impression safely. The 3D-scanning technique presented the opportunity to obtain a deeper scan of the ear canal to create an earmold to keep the ear canal open. This patient only underwent the 3D-scanning procedures in our clinic. Performed by the experienced audiologist, 3D-scans were taken of both ears, the patient was fit with the earmolds, and the outcomes were followed and compared.

## 3. Results

The objective results and subjective responses from the participants and the audiology professionals were recorded. Multivariate analysis of variance (MANOVA) with repeated measures was used to compare the differences between these two techniques and between the groups of participants. A post hoc test was used for further individual category response comparisons. The alpha level for significance was *p* < 0.05. Descriptive analysis was also used for different comparisons.

### 3.1. Impression Technique Comparison

#### 3.1.1. Participants’ Perceptions

**Figure 2:**
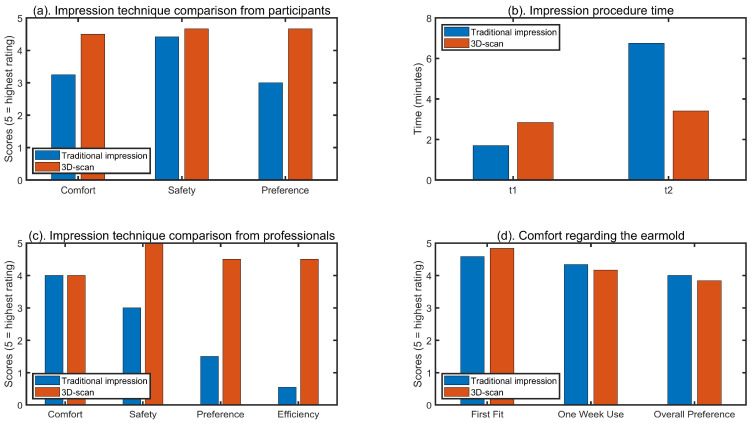
Comparison results of impression technique, earmold comfort, and procedure time.

The bar graph in Figure 2a shows the average response results from the participants regarding the two techniques. The 3D-scanning technique was rated higher in feelings of comfort, slightly higher in safety, and participants preferred 3D-scanning. The across-technique analysis from MANOVA revealed F(1,21) = 0.46, *p* = 0.006, suggesting that there was a significant difference among the responses for the two techniques. Post hoc tests suggested significant differences in the category of feeling of comfort (*p* = 0.01) and technique preference (*p* = 0.003), but not for the safety (*p* = 0.48). The across-group analysis revealed F(1,21) = 0.08, *p* = 0.21, suggesting that there was no significant difference among the responses from the two groups (earmold-users vs. non-users). Table 2 includes more detailed survey results from the participants. The mean and median were higher for the 3D-scanning technique. There was a wider range and more variation in the scores for the traditional impression than the 3D-scanning technique for both groups.

#### 3.1.2. Time for Impression Techniques

Figure 2b shows the average time per ear taken for each technique by audiology professionals. The average procedure time was shorter for T1 than 3D1, but longer for T2 than 3D2. The across-technique analysis from MANOVA revealed a significant effect of technique on time spent (F(1,22) = 0.21, *p* = 0.045) and a significant interaction effect of the procedure and technique on time (F(1,22) = 3.88, *p* < 0.0001). Post hoc tests suggested significant differences in T2 vs. T1 (*p* < 0.0001), T2 vs. 3D1 (*p* < 0.0001), T2 vs. 3D2 (*p* < 0.0001), and 3D2 vs. T1 (*p* = 0.02) but not for 3D1 vs. T1 (*p* = 0.2) and 3D1 vs. T1 (*p* = 0.75). The across-group analysis revealed F(1,10) = 0.93, *p* = 0.001, suggesting that there was a significant difference between the impression time for the two audiology professionals. Further comparison revealed no significant difference in T1 (*p* = 0.43) and T2 (*p* = 0.84) between the two professionals, but the young clinician performed faster compared to the experienced audiologist when using 3D-scanning for both 3D1 (*p* = 0.002) and 3D2 (*p* = 0.0005).

Table 3 includes more details on impression time spent for the two techniques by the audiology professionals. The procedure time per ear was shorter for T1 (1.71 min)/3D1 (3.98 min) and longer for T2 (6.21 min)/3D2 (4.85 min) for the experienced audiologist. For the young clinician, the procedure time per ear was similar for T1 (1.68 min) and 3D1 (1.70 min) but was longer for T2 (7.23 min) compared to 3D2 (1.96 min). The average time differences (T2–T1)/(3D2-3D1) were 4.5/0.87 min for the experienced audiologist and 5.61/0.26 min for the young clinician, indicating longer times for traditional impressions for both professionals. There was a wider range and more variation in the overall procedure time spent (t2) on the traditional impression than the 3D-scanning technique for the young audiology professional.

#### 3.1.3. Audiology Professionals’ Perceptions

Figure 2c shows the survey results from the audiology professionals regarding the two techniques in four categories including comfort in administering the technique, ensuring the safety of the patient, overall preference for technique, and clinical efficiency. Results revealed similar ratings between the techniques in the category of comfort in administering the technique, and higher ratings for the 3D-scanning technique in the other three categories. Both professionals had similar ratings, except that the experienced audiologist felt more comfortable conducting traditional impressions while the young clinician felt more comfortable with 3D-scanning. On the question of “How long you feel it takes/would take to master executing this technique for ear impressions?”, the answers from both audiology professionals indicated approximately 10 h of training including some hands-on practice to achieve a basic understanding of traditional impression, and longer training for proficiency. For the 3D-scanning technique, both audiology professionals suggested less training is needed to achieve proficiency, including two to three hours of training and some practice on real subjects.

### 3.2. Earmold Comfort Comparison

The average questionnaire results regarding the perceived comfort of earmolds made from both techniques can be seen in Figure 2d. Results revealed a slightly higher rating for the earmolds made from 3D-scans at the first fit but higher after one week of wear for those made from traditional impressions. The participants had a slightly higher preference for the earmolds created by traditional impressions. The across-technique analysis from MANOVA revealed F(1,10) = 0.0003, *p* = 0.96, suggesting that there was no significant difference among the responses between the two techniques. Table 4 includes more detailed survey results of earmold comfort. The mean was similar for the techniques (0.25 difference between scores for first fit and approximately 0.17 difference for one week of wear and overall preference). There was a wider range and more variation in the scores for the traditional impressions than 3D-scans for the first fit, but the opposite for one week of wear and overall preference.

### 3.3. Earmold Cost and Time Efficiency

Ear impressions are needed to make custom earmolds to fit each individual’s ears. For traditional ear impression, materials include those with fixed costs/one-time investments (otoscope, speculum, syringe (with spatula)/impression gun, otolight + tip) and others with variable/recurring costs (impression material/cartridge, splead pads, otoblocks, alcohol wipes, shipping box). For 3D-scanning, the materials include a one-time investment in a 3D-scanning set (a computer + scanning set) and otoscope + speculum, and variable costs of alcohol wipes. The earmold price will be the same from both techniques. The fixed costs are significantly higher for a 3D-scanning set compared to that of traditional impressions. The variable costs per earmold are approximately 7% less for a 3D-scan compared to the traditional impression. In addition, there is only half the shipping cost and time associated with 3D-scanning due to one less shipment, achieved by the scan being transmitted to the Otocloud (online database for Otoscan device). The manufacturer collects the 3D-scan from Otocloud instantaneously to create the earpiece, which is then shipped to the clinic. With the traditional technique, the impressions must be mailed to the manufacturer to create the custom earpiece and then mail it back.

### 3.4. Subjective Feedback from Participants

Regarding the impression techniques, the participants felt that the 3D-scanning impressions were more comfortable and found the process interesting to watch; they felt that the traditional impression material made their ears very full, cold, and uncomfortable. Regarding the earmolds created from 3D-scans, they felt that the earmolds were less thick, not as uncomfortable/tight, and easy to remove, but that they allowed some acoustic feedback and created uncomfortable feeling at helix or behind tragus. Regarding the earmolds created from traditional impressions, they that felt the earmolds did not or did create pressure/full feeling in the canal, had a good fit, did not cause any acoustic feedback but seemed harder to put in the ear.

### 3.5. Special Clinical Case Results

**Figure 3:**
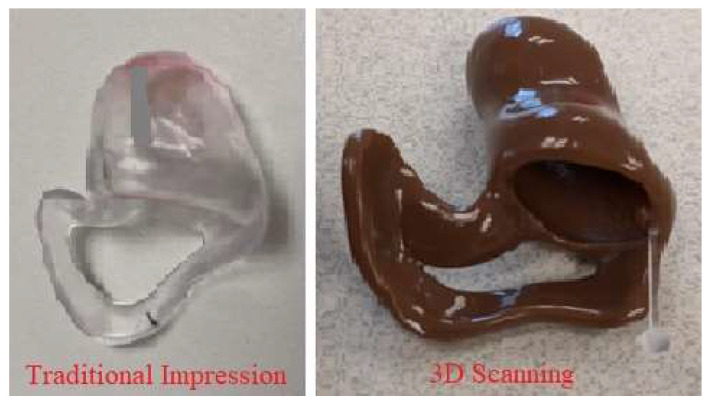
The earmolds of the special case created from two techniques.

Regarding the special clinical case, earmolds created by the 3D-scanning technique were favored and provided better benefit. The hollow earmolds created from traditional impressions were not long or deep enough in the ear canal (Figure 3). Therefore, the tissue beyond the earmold continued growing shut and conductive hearing loss consistently reoccurred. The 3D-scanning technique allowed for a deeper scan to be obtained safely and further created an earmold that is much longer and deeper in the patient’s ear canal (Figure 3). These new earmolds created by 3D-scans have been successful in keeping the entire ear canal open and not allowing any skin closure beyond the earmold. This has prevented conductive hearing loss from reoccurring, and reduced the need for additional surgeries to re-open the canal.

**Figure 4:**
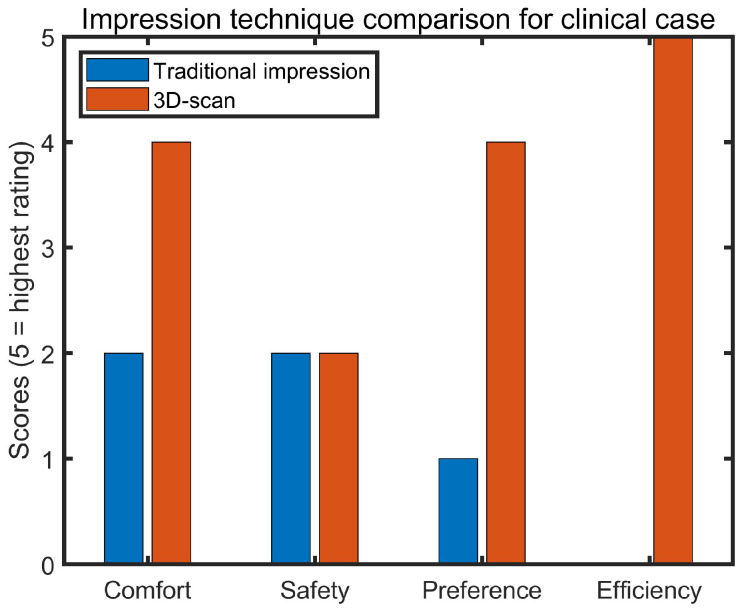
Impression technique survey results from the experienced audiologist for the clinical case.

Figure 4 shows the preference survey results of the experienced audiologist in terms of the two techniques for the special clinical case. The results revealed higher ratings for 3D-scanning in the category of comfort in administering the technique, overall preference for technique, and clinical efficiency. Although they rated both techniques the same in the category of ensuring the safety of the patient, the audiologist commented that “I feel very uncomfortable inserting foreign objects such as otoblocks and impression material in ears with known outer ear infection”, and “My only concern regarding use of the 3D-scanning technique was patient safety. The child sat still but had to be encouraged to do so and this would have been the same for traditional techniques.”

## 4. Discussion

### 4.1. Impression Technique Comparison from the Participants

Results revealed a significant effect of the technique on the responses. The participants felt significantly more comfortable with the 3D-scanning technique and preferred this technique in comparison to the traditional technique. They felt that it was more comfortable as their ears were not plugged with the silicone material and they enjoyed seeing the video of their ear canal on screen. As seen in Table 2, there was a wider range and more variation in rating scores for the traditional impression technique compared to the 3D-scanning technique for both groups of participants. Some young adult participants rated ‘0’ or ‘1’ for the traditional technique, suggesting they felt very uncomfortable with the technique. Even for participants with the experienced audiologist, some did not feel comfortable and did not like the traditional technique, giving a rating score of ‘2’ in comfort and preference, and ‘3’ in safety, implying they were unsure about the safety of the traditional technique. Although, overall, the 3D-scanning technique was rated slightly higher than the traditional technique, there was no statistical difference between the two techniques in the category of safety. Since participants feel more comfortable with and prefer undergoing the 3D-scanning technique, this will lead to greater patient willingness to have scans taken of their ears. In turn, this leads to more custom earmolds being made, therefore yielding better hearing aid fitting outcomes for patients needing a custom-fit instead of non-custom domes.

### 4.2. Earmold Comfort Comparison

Feedback regarding the comfort of earmolds revealed no significant effect of impression techniques, which indicated that both techniques are sufficient in creating comfortable custom ear products. There was one participant who gave a rating of ‘2’ after one-week use and ‘0’ for overall preference for the earmolds from 3D-scans due to an uncomfortable feeling in the helix of the patient’s right earmold, which made the average 3D-scan scores slightly lower than those from the traditional technique. Note that the earmold was not modified for this experiment, while audiology professionals will typically modify the earmold to solve the fit problem. In general, most of the participants felt comfortable with the earmolds from both techniques, although there were some different issues present for each technique. For example, there was pressure or a fullness issue in the ear canal for the traditional technique, as well as acoustic feedback and possible discomfort at the helix or behind the tragus for the 3D-scanning technique. Silicone, a common impression material, was used in this study, which will stretch the ear canal due to its high viscosity property [12,27,28] and, hence, may cause the fullness feeling or too much pressure in the ear canal from the final earmold product. In contrast, making impressions using the 3D-scanning technique will scan the surface of the ear including the ear canal while making no contact with the ear, which may cause feedback from the final earmold due to a possible loose fit, especially for those with a flaccid or soft ear texture and high-gain hearing aids. Clinically, listeners who require more amplification (e.g., severe to profound hearing loss and mixed hearing loss) would need a tight fit for the earmold to prevent feedback, for which a traditional impression might be a better choice. For 3D-scans, a note might be suggested to the product team in the order form to indicate the need for a tight fit for the patient. Future studies could recruit listeners with different types and degrees of hearing loss to compare the hearing loss effects on the perception of the impression techniques. Due to anatomical position, especially for the helix portion, there may be technical difficulty in scanning and this can cause the earmold to feel uncomfortable. The similarity in the perceived comfort of both techniques allows clinicians to be confident in the final product created. Knowing that the earmold or custom hearing aid will provide good fit and sound quality for patients from either technique allows clinicians to rest assured that their patients are receiving great benefit from their hearing aids or other custom earpieces.

### 4.3. Impression Technique Comparison from Audiology Professionals and Clinical Efficiency

The average results from the audiology professionals revealed similar ratings between the techniques regarding comfort in administering the technique. However, the individual responses revealed that the experienced audiologist felt comfortable with both techniques but more comfortable with the traditional technique (rating 5 for traditional, 4 for 3D-scan), while the young clinician felt comfortable with the 3D-scans and less comfortable with the traditional technique (rating 4 for 3D-scan, 3 for traditional). Experience with the techniques may play a role on these ratings. Although both audiology professionals had the same training on the 3D-scanning technique (less than a month with a few hours of training and a few practice subjects), the experienced audiologist had more experience with the traditional technique (over 18 years). The young audiology clinician was a second-year Doctor of Audiology student and the total training hours and practice subjects were similar for both techniques; though they had more exposure to the traditional technique including two hearing aid courses with the introduction of the traditional technique, two clinic practicum courses and one summer clinic with observations of the traditional technique before this project. It seems that mastering the 3D-scanning technique can be achieved faster than mastering the traditional technique, though more training will bring a more positive response in terms of skills. This project only included two audiology professionals; future studies could include more audiology professionals to determine the experience effect on the skills of administering the techniques.

Both audiology professionals felt safer in administering the 3D-scans (rated 5) than the traditional technique (experienced audiologist rated 4, young clinician rated 2) because there is no contact with the patient ears for 3D-scans. Experience with the impression technique may have played an effect on the responses. The experienced audiologist, with more experience in impression techniques in general, felt safe using both techniques but safer with the 3D-scanning technique (rating 4 for traditional, 5 for 3D-scan); the young clinician with less experience on impression techniques in general felt safe using the 3D-scans and unsafe using the traditional technique (rating 4 for 3D-scans, 2 for traditional). Both audiology professionals did indicate the need for more experience to be proficient in the traditional technique, which may provide an explanation for the responses from the young clinician. Traditional impression techniques have been used in audiology clinics daily for a long time. Proper insertion of the size-appropriate otoblock and being well trained in the impression injection technique should yield a safe and good impression product. Limited experience in the traditional technique may have resulted in the young clinician rating a ‘2’ for this technique.

Both audiology professionals felt the 3D-scan is more preferable and clinically efficient. One reason for this is that obtaining scans of the ear is more time efficient. As indicated in the above result section, both clinicians took less time to complete one impression for 3D-scans (young: 1.96 min; experienced: 4.85 min) when compared to the traditional technique (young: 7.29 min; experienced: 6.21 min). With practice, the 3D-scan is expected to take approximately 2 min per ear, cutting out the time that the materials for traditional impressions requires to cure after being delivered into the ear canal. Making earmolds via 3D-scanning also needs half of the shipping time, considering it directly uploads the scan to Otocloud while the traditional technique requires shipping of impressions to the manufacturer. This allows more patients to be seen in a day and more hearing aids being fit in a clinic. Additionally, making earmolds using 3D-scans may be cost effective, with approximately 7% lower variable costs per earmold due to lower material costs compared to the traditional impression, if not considering the fixed costs which are significantly higher for the 3D-scan. Clinics should consider their own budget and try to bring down the cost to promote custom earmolds for patients. This, in conjunction with the comfort and confidence of professionals in being able to create a custom earmold, will further lead to better hearing aid fitting outcomes for patients needing a custom fit.

### 4.4. Special Clinical Case

The special clinical case study indicated the medical necessity of using 3D-scans for some special cases to achieve the best clinical outcomes. The 3D-scans allowed for deeper impressions to create a longer canal without needing physical contact with the ear. This assisted in preventing this patient from experiencing recurrent conductive hearing loss as well as from reducing the number of additional surgeries to re-open the ear canals. Some other clinical cases that may consider using 3D-scans include people with sensitive ear canals, surgical ears such as mastoid cavities, individuals on blood thinners, people who are uncomfortable with having impression material inserted into their ear canal, and cases that require deep ear impressions. Note that the scanning tip is hard and approximately 3 mm in diameter, which is not appropriate for very small ear canals, hard and curvy ear canals, and those who cannot sit very still for approximately 2 min. Under these circumstances, the traditional technique should be administered. As indicated in the result section, the time from the end of otoscopy to the end of impression material delivery was less than 2 min for both audiology professionals, which includes placing the otoblock and the delivery of the impression material into the ear canal. Therefore, the time to inject the impression material into the canal may be less than 1 min. For older children with big enough ear canals and those who can sit still for the procedure, with consent from both the child and the parent, the 3D-scanning technique could be an option. Children’s ear canals are of adult size (approximately 2.5 to 3 cm long, 0.75 cm in diameter) by 7–10 years of age but can vary from case to case [29,30]. Also, children four years and older might be able to sit still for the impression process.

## 5. Summary and Conclusions

The results of this study revealed that the participants perceived both techniques to be safe, but felt more comfortable with and preferred the 3D-scanning technique compared to the traditional technique. The 3D-scanning procedure provides an engaging experience as it allows the patients to watch the scan being created on the computer screen. The participants considered the earmolds generated from both techniques at a similar level of comfort and with no significant preference for either of the earmolds, although the earmolds may have a tight fit from traditional impression and loose fit or feedback and helix/tragus issues from 3D-scans. Different types and degrees of hearing loss as well as ear texture may need to be considered when choosing the appropriate impression technique for each individual for a better fit (e.g., a traditional impression with a tight fit might be better for mixed or more severe hearing loss). Regarding the perception of the techniques by the audiology professional, experience with the impression technique may have played an effect on the responses. The experienced audiologist, who had more experience with the traditional technique, was comfortable with both techniques and felt safe using both techniques. The young audiology clinician felt more comfortable with 3D-scans and felt safer utilizing the 3D-scans despite the similar level of training experience. Also, it seems faster to master the 3D-scanning technique compared to the traditional one. More audiology professionals should be included in future studies to confirm the experience effect on the techniques. Both audiology professionals preferred 3D-scans and considered it to be more efficient clinically due to time and possible cost efficiency. In addition to the 7% less variable costs per earmold, the time taken for the impression procedure and earmold production and the shipping cost for the 3D-scan is half that of the traditional impression, though the fixed costs are significantly higher for 3D-scans. The special clinical case indicated that 3D-scanning can be a better solution compared to the traditional impression considering specific situations, which required deep ear impressions in this case. Other situations could be those with a sensitive ear canal, surgical ears with an abnormally large canal volume, patients on blood thinners, etc. Note that 3D-scanning is not appropriate for very small, hard, and curvy ear canals, and those who cannot sit still for approximately 2 min. Both techniques have their merits and can be used for different populations on a case-by-case basis.

In conclusion, this study provides good insight regarding multiple clinical aspects of 3D-scanning and traditional ear impression techniques, and leads to further and better understanding of what technique serves as the better practice protocol. Both techniques are safe and can produce comfortable earmolds. The implementation of 3D-scanning will increase clinic efficiency as the variable costs and time needed are decreased with the scan technology and may provide a more positive and comfortable experience for patients. With increasing involvement of new technology and more young professionals joining the profession of audiology, the 3D-scanning technique would be a good addition for an audiology practice.

### Limitations of This Study

This study had a small participant pool including 12 adult participants (6 earmold uses, 6 non-earmold users) and one special clinical case, which cannot be generalized to all patient populations. In addition, two audiology professionals with different experience in impression techniques participated in this study. Also, they completed the impression techniques on different groups of subjects, and only the earmold-user group experienced earmolds from both techniques. Future studies should recruit a larger participant pool with different types and degrees of hearing loss to address individual differences, as well as including more audiology professionals with different impression technique experiences.

## Figures and Tables

**Table 1:** Background information of participants with earmolds.

Subject #	1	2	3	4	5	6
Age and Gender	67 years, M	68 years, M	62 years, M	62 years, M	53 years, F	58 years, F
Hearing Loss	R: WNL-Pro SNHL, L: WNL-Se SNHL	R: WNL-MoSe SNHL, L: Mi-MoSe SNHL	Mi-Se SNHL, Au	Mi-Se SNHL, Au	R: Mi-Pro SNHL, L: Mi-Mo SNHL	MoSe-Pro MHL, Au
HA History	Since 2013	Since 2014	Since 2015	Since 2014	Since 2008	Since 2015
Earmold Style	Skeleton	Skeleton	Skeleton	Skeleton	Full shell	Full shell
Material	Acrylic	Acrylic	Acrylic	Acrylic	Silicone	Acrylic
Vent	Large	Medium	Small	Small	R: Pressure L: Medium	Pressure
Canal Length	Medium	Medium	Medium	Short	Short	Medium
Tubing	Thick	Medium	Thick	Medium	Medium	Thick

Note: HA = hearing aid; WNL = within normal limit; Mi = mild; Mo = moderate; MoSe = moderately severe; Se = severe; Pro = profound; Au = both ears; SNHL = sensorineural hearing loss; MHL = mixed hearing loss; F = female; M = male; L = left; R = right.

**Table 2:** Impression technique survey results from the participants.

		Comfort		Safety		Preference	
		T	3D	T	3D	T	3D
Earmold Users	Mean	3.6667	4.6667	4.6667	4.8333	3.3333	4.6667
	Median	4	5	5	5	3.5	5
	Min	2	4	3	4	2	4
	Max	5	5	5	5	5	5
	STD	1.3663	0.5164	0.8165	0.4082	1.2111	0.5164
Non-earmold Users	Mean	3	4.6	4.6	4.8	2.4	4.8
	Median	3	5	5	5	3	5
	Min	1	4	4	4	0	4
	Max	5	5	5	5	4	5
	STD	1.4142	0.5477	0.5477	0.4472	1.8166	0.4472

0–5 scale (5 = the highest rating); T = traditional impression; 3D = 3D-scanning; STD = standard deviation.

**Table 3:** The time to take impressions/scans from audiology professionals.

		t1		t2	
		T	3D	T	3D
Experienced Audiologist	Mean	1.7111	3.9819	6.2139	4.8542
	Median	1.6	3.5292	6.2292	4.725
	Min	1.2667	2.9333	5.5833	3.5583
	Max	2.0383	5.6667	7.0917	6.375
	STD	0.416	1.1152	0.5376	1.0654
Young Clinician	Mean	1.675	1.6972	7.2889	1.9556
	Median	1.625	1.6667	6.6708	1.9042
	Min	1.375	1.5083	5.3167	1.775
	Max	2.1	1.975	11.8667	2.1917
	STD	0.3111	0.1839	2.3227	0.1733

t1 = time from the end of otoscopy to the end of impression material delivery/scan; t2 = time from the end of otoscopy to the end of impression removal and inspection/scan inspection; T = traditional impression; 3D = 3D-scanning; STD = standard deviation.

**Table 4:** The earmold comfort survey results.

	First Fit		One Week Use		Overall Preference	
	T	3D	T	3D	T	3D
Mean	4.5833	4.8333	4.3333	4.1667	4	3.8333
Median	5	5	5	4.5	4	5
Min	3.5	4	3	2	3	0
Max	5	5	5	5	5	5
STD	0.6646	0.4082	1.0328	1.169	0.8944	2.0412

T = traditional impression; 3D = 3D-scanning; STD = standard deviation.

## Data Availability

All data generated or analyzed during this study are included in this article.
